# A “Hijacked Salaried Status” in French Cooperatives of Freelance Workers: The Political Meaning of Shifts Between Standard and Non-standard Employment

**DOI:** 10.3389/fsoc.2020.00036

**Published:** 2020-05-27

**Authors:** Flora Bajard

**Affiliations:** Aix Marseille Univ, LEST (UMR 7317), Marseille, France

**Keywords:** self-employment, wage-labor, business and employment cooperative, indefinite employment contract, entrepreneur, non-standard employment, politicization, institutional change

## Abstract

This article considers a specific sub-type of non-standard employment—self-employment—through a particular type of cooperative in France: the Business and Employment Cooperatives (BEC), i.e., collectives of freelance workers. BECs aim to provide an indefinite employment contract—and the social protection associated with it—to these individuals who therefore become “salaried entrepreneurs.” To better understand the gray zones of work, where legal status, practices and identities are often disconnected, this inquiry is based on a qualitative approach to social actors' practices. It shows, on a meso level, how BECs “hijack” the standard wage-labor contract on the grounds that this is emancipatory and therefore drag it into a non-standard form of employment. In addition with this first shift between the legal framework and its interpretation, a second shift occurs as each member of the cooperative—a “false wage earner”—develops a singular relationship to the constraints related to such a contract. Through the notion of “praxis,” combining both objective and subjective dimensions of work, we are able to systematize the analysis of qualitative data and identify the factors that influence such a diversity of appropriations: the relationship to conflictuality and political competence. Finally, this article highlights the conditions under which social actors make a wide range of appropriations of common legal frameworks, whose flexibility requires us to consider employment relationships as variant and creative practices rather than as “perfect” or “deviant” forms.

## Introduction

[We see] “a second way to the universal: no longer the overarching universal of a strictly objective method, but a sort of lateral universal which we acquire through ethnological experience and its incessant testing of the self through the other person and the other person through the self” (Merleau-Ponty, in Bachir Diagne, 2013, p. 16).

This philosophical issue, rooted in a very different context, inspires us to examine the ties between the standard employment contract^1^ and its non-standard variants^2^. Beyond the formal opposition between both forms, this article addresses the two kind of shifts that arise between standard and non-standard forms of work within a single legal framework—the indefinite employment contract. In order to do so, one has to consider not only “employment” conditions but also work in its very practical dimension, through the reality of workers' living and working conditions. Studying the “gray zones” of work may demonstrate the accuracy of this approach in a striking way as the main characteristic of the gray zones is to blur the traditional lines that usually help to describe the types (and sub-types) of employment.

Concerning independent work, which is at the core of this article, the frontier between wage labor and self-employment has contributed to the definition of the standard employment relationship (a full-time and indefinite employment contract) during the twentieth century^3^. Yet, some important evolutions have occurred within this dichotomy, as French jurist Supiot (2000) demonstrated. These transformations have ended dualist labor approaches (insiders vs. outsiders of labor markets; wage-earning job vs. self-employment, i.e., standard and non-standard employment relationships). This expanding complexity of social and professional patterns^4^ has therefore led to a structural phenomenon of “decoherence” of employment standards: “The proliferation of areas of lawlessness and legal confusion, [and] also strategies for circumventing and gaming the multiplicity of existing rules” (Bureau and Dieuaide, 2018, p. 263). A gray zone can be understood as a public space where there is a “mediation between ‘intangible’ rights and duties a priori guaranteed by labor law, on the one hand, and crumbling interests that escape a priori from any procedural rationality, on the other hand. … [It is a space] where balances are created by hybrid and implicit rules or non-constraining conventions (soft law). As a public space, a gray zone is a more or less informal deliberative space that combines, or even mixes, two different orders of legitimacy, in practice: one comes from institutions and the State, the other from the market and contractual reasoning” (Azaïs et al., 2017, p. 442)^5^.

Thus, the issue of gray zones of work and employment are of high interest to sociologists as they suggest that formal limits and definitions are not sufficient to comprehend the current challenges raised by these evolutions. Therefore, we must particularly consider the *reality* of work if we now want to understand this. Not that we totally relativize the importance of objective frameworks, such as economic and legal constraints, but we assert that neither organizations nor statuses/standards of employment fully determine workers' living conditions. This article is focused on this line of argument and uses the notion of “praxis,” which aims to comprehend “work” through its *practical* dimension. The idea of praxis refers to the production of the self through labor in a unified conception of representations and practices. From this perspective, labor is not only the externalization of consciousness of the world but it can also be understood as a practical worldview or the constant daily practice of one's worldview. The notion therefore embraces objective constraints and subjective representations, and enables us to address the articulation between institutional frameworks and individual action at work (Dardot, 2015).

This first argument feeds a second, which implies distancing oneself from the normative approaches of the gray-zone phenomenon. This phenomenon often signifies a regression which may reverse the “forces of emancipation” (Bureau and Dieuaide, 2018, p. 275) and these transformations have taken place in a general context of the weakening of the social protection and salaried norm. Although wage labor still represents a major fraction of total employment, the standard employment relationship of the indefinite and full-time labor contract is decreasing through several processes: new statuses (0-h contracts, micro-entrepreneurship), technological changes (platform and gig economy), and ideological changes in collective and individual preferences and expectations (flexibility, autonomy, incentives for self-employment, etc.). The case of the solo self-employed offers an interesting insight into these trends. For example, some employment policies in Western Europe encourage people to consider the possibility of becoming a freelance worker, sometimes even part-time. Some legal statuses, like the “economically dependent autonomous worker” in Spain and the “auto-entrepreneur” in France, allow for more flexibility and pluriactivity. However, they also generate a greater precariousness^6^; one of the main illustrations of this is the bogus self-employment which proliferates throughout uberization processes. In self-employed relationships, we may therefore talk about the “self-employed precariat,” which is “reflective of complex and diverse patterns of atypical work that is growing, ranging from casual working to temps, agency staff, own account workers and Uber drivers. The self-employed precariat do not enjoy employment rights and protections at work, or any of the implicit services associated with being an employee, such as payroll or workplace insurance—let alone pension or sick pay” (Conaty et al., 2016). To some extent, the many new solo self-employed resemble the workers of the nineteenth century, selling their labor on a day-to-day or piecework basis, and this is why many researchers often refer to the gray zones of employment through the precariousness matrix^7^. However, it also seems necessary to seriously consider the “various sub-types of non-standard employment” as bogus/dependent self-employment is far different from other kinds of solo self-employment (Hipp et al., 2015, p. 368). Besides, along with other observers^8^, we argue that gray zones should not *necessarily* be considered a “shadowy part” of labor markets nor as chaotic situations for lacking political regulation (for developments on the same line of argument, see, for example, Bureau and Dieuaide, 2018, p. 262–265). In fact, the non-standard employment relationship should not automatically be considered as a “lack” or a “failure” *per se*, as some political and institutional alternatives and responses also occur in the workplace. Rather, in this article, we wish to point out the ambivalence of these gray zones, their openness, and absence of institutional determinism that allows us to address the appropriation of such organizations by the actors. This article is focused on presenting arguments that explore the following three ideas: considering the reality of work, identifying sub-types of employment, and avoiding normative labels of categories of work and positions.

To this aim, the text reveals the findings of a study of a particular type of cooperative in France: Business and Employment Cooperatives (BECs), which can be described as collective groups of freelance workers^9^. Today, there are around 100 BECs in France, which represent around 10,000 freelance workers in total^10^. They are a very particular form of the gray zone of work which are thought by its promoters to be institutional innovations and responses to the precariousness and difficulties of the self-employed. The BEC includes different kinds of freelance workers and professionals who maintain their autonomy, while working under an indefinite employment contract and receiving a wage from the cooperative—with the social protection it provides. In other words, they benefit from the social protection of the wage-labor status, while avoiding the difficulties of self-employment: loneliness, weaker social protection, income discontinuity, etc. At the same time, they are not subordinate to any employer. This subordination which forms the basis of wage labor is at the core of this paradoxical status: they are “autonomous employees” (Grégoire and De Heusch, 2016), officially called “salaried entrepreneurs” since the Hamon law of 2014.

In the first part of this article, we show that the standard employment norm—wage labor and its indefinite employment contract—experiences a double “shift” in the BEC. The first type of shift occurs between the institutional rules of the standard employment relationship and the organizational rules of the BEC, which are created by “hijacking” the salaried contract. The second type of shift is developed through actors' individual practices via which they individually appropriate this already displaced and altered form of contract. Gradually, social actors'—whether they are BEC staff or entrepreneurs—practices create a diversity of shifts in comparison with the initial rules of the indefinite employment contract.

## Materials and Methods

In 2018 and 2019, we carried out in-depth interviews^11^, with an average time of 2 h 30 each, with 16 entrepreneurs from three different BECs in France. We also interviewed 5 project managers from three BECs, as the permanent staff of these organizations. We complemented these interviews with observations made during events that brought together BEC representatives and members, as well as a few observations of building workers (masons, electricians, etc.) during their ordinary professional practices. The occupations of our interviewees come from very different sectors and positions within the social strata, as shown in Table 1. In addition, the situation of these entrepreneurs is quite different in terms of income (some barely survive with the minimum state insurance, whereas others, like Pierre, estimate their monthly net income to be more than 3,000€. Some depend on very few clients, like Eymeric who relies on a couple of contractors and rarely manages to win contracts with new customers, while others have multiple income sources, like Maïwenn who mixes training, individual therapy, and workshops. Also, some of them are project leaders, like Jeremy who has a genuine and wide customer portfolio and sometimes even sub-contracts part of his building works to his colleagues, whereas others have a very small professional network and rely on their colleagues to make their activity financially viable. In other words, our interviewees have very different power-relation positions within the workplace. Table 1 shows the main characteristics of the entrepreneur interviewees of our sample (not the BEC representatives and staff). The names of all interviewees and organizations have been changed to protect their anonymity.

**Table 1:** Interviews with French entrepreneurs in 2018–2019.

**Name**	**Age**	**Sex**	**Organization and duration of membership**	**Occupation**	**Type of employment contract in the BEC at the time of the interview (see explanations in part 3.1)**
Eymeric	46	M	Alpha (2 years)	Photographer	CAPE
Pauline	29	F	Alpha (2 years and 3 months)	Web writer	Associate entrepreneur
Jeremy	30	M	Omega (2 years)	Mason	Associate entrepreneur, working with an associate partner (unique situation within the BEC)
Maïwenn	56	F	Omega (2 months but has previous experience in other BEC)	Non-violent communication therapist	CAPE
Samia	36	F	Omega (3 months)	Art therapist	CAPE
Pierre	57	M	Omega (6 months)	Innovation and strategy consultant for small businesses	CESA
Lev	38	M	Omega (2 years and 1 month)	“Green” real estate agent	CAPE
Jean-Philippe	39	M	Omega (18 months)	Electrician	CAPE
Gary	33	M	Alpha (10 months)	Researcher in computational languages	CAPE
Saïd	34	M	Alpha (13 months)	Artificial Intelligence researcher and consultant	CAPE
Dounia	50	F	Omega (between 1 and 2 years)	NGO and small businesses consultant	CAPE
Denis	51	M	Alpha (2 years and 9 months)	Consultant in strategy (environmental and social communication)	Associate
Lila	**31**	**F**	Omega (3 years, and then quit)	Communication expert and consultant	CESA
Leïla	35	F	Beta (2 years)	Sociologist	CESA
Maëlis and Vincent	26 and 28	F and M	Alpha (about 1 year and a half)	Dog instructors	CAPE

The three cases used for this article are highlighted in gray

This article is mainly based on the interviews that we conducted with freelancers, as the political management of the BEC is not at the core of this paper. The interviews were carried out at interviewees' homes or sometimes in other places, such as cafes or co-working spaces that some use as their personal office. Almost all interviews took place within the area of an important city in Southern France. The conversations included not only their job and career but also the private aspects of their lives, including their childhood, hobbies, way of life, and family life. The qualitative approach of our research enabled us to carry out a very deep analysis of the interviewees' social pathways and situations (Table 2). The disadvantage of this approach is that we lack space here to extend the examples and individual case studies. For methodological reasons, and in order to provide a structured and unified case study, we therefore base the second part of our article on three individuals. The three cases analyzed here correspond to freelance workers who all belong to the same organization; when they refer to their BEC, they are therefore speaking about a single cooperative. However, the findings of our study are based on the whole sample of our research, and we selected these three cases to exemplify and provide evidence based on their diversity and illustrative capacity.

**Table 2:** The analytical framework elaborated from the notion of praxis in order to proceed the collected data.

**CONCEPTIONS OF… (subjective dimension)**		**CONDITIONS (objective dimension)**		
*Wage-earning*		*Economic and social*	Personal life	
*status*		*background*	Income	
*Self-employment*			Patrimony/heritage	
		*Position within power relations*	At the workplace	
*Cooperation*			Political/civic/trade union commitment	
		*Working conditions*	Use (or not) of social protection	
*Political views/conflictuality:* -*About society* -*At the workplace*			Position within the cooperative When self-employed When a wage earner	

## Results and Discussion

### The “Hijacking” of the Salaried Contract Within “Cooperatives of Freelancers”: a First Shift Between Standard and Non-Standard Employment

Unlike the traditional model of agricultural or industrial cooperatives, in which all workers produce a common product or service, the BEC gathers different professional activities: for example, in one single BEC, there may be a gardener, an architect, an artist, a management consultant, etc. Above all, the BEC represents one of the highest levels of cooperativism: they are enterprises in which autonomous workers are salaried employees. In fact, some freelance cooperatives only offer shared services, for example, they mutualize tasks or competencies to market their services to customers, or they mutualize a common working-place, but their members remain genuinely self-employed since each of them is paid by his/her contractor. Instead, in the BEC, self-employed people form cooperatives for shared services and also get paid by the cooperative, which produces invoices for their contractors and pays each member a wage. The members of the cooperative are therefore no longer genuinely self-employed workers although they consider themselves to be freelance because they work autonomously and dedicate themselves to their own personal project. Also, each of them has a wide autonomy within the different stages and dimensions of his/her work: the entrepreneurs decide how much they plan to earn, on which days and at which times, where and with whom they wish to work (whether they are clients or colleagues), and which strategy to develop to improve their competencies, etc. They are also entirely responsible for the economic viability of their activity. As the French Labor Code underlines, the salaried entrepreneurs are employees of the BEC, which is therefore responsible for their state insurance contributions and information about their health and working conditions^12^. However, entrepreneurs are given a high level of autonomy in their work, which also means less security. This flexibility and low level of support from the BEC is sometimes criticized by some entrepreneurs (see Bajard and Leclercq, 2019). In spite of these autonomous conditions, all entrepreneurs share the same company registration (SIRET) number and their individual sales generate a common sales revenue that makes the cooperative financially viable^13^. This enables, for example, the cooperative to employ a staff of people dedicated to administrative and organizational tasks, such as communication, accounting, etc. Finally, each freelance worker also may become a full member of the cooperative, which means that he/she belongs to a collective entity that relies on organizational and democratic rules and shares the added value generated within the cooperative. This is one of the main differences from “umbrella companies,” which do not intend to implement cooperative principles and rules.

Three different steps constitute the classical “salaried-entrepreneur” career within a cooperative. The CAPE contract—*Contrat d'Appui au Projet d'Entreprise* (Support Contract for the Business Project)—enables any member to combine income from his/her activity with a part-time job in a company or with unemployment benefits (or other State income). During that period, he/she capitalizes part of this income in a personal account within the BEC. When the professional activity is “on track” and the member has enough funds to steady and homogenize the income he/she has capitalized, he/she is then able to pursue the activity under a CESA contract—*Contrat d'Entrepreneur Salarié Associé* (Contract of Associate Salaried Entrepreneur, an indefinite employment contract)—which is the “core step” of the BEC model. For a freelance worker, working under this contract means officially becoming an employee of the cooperative. However, his/her wage depends on the volume of incomes capitalized in his/personal account and is defined by a contract that may be revised depending on the evolutions of the activity (see below). The last step consists of choosing whether or not to become an associate of the cooperative^14^. Eventually, these freelance workers avoid the difficulties of self-employment (loneliness, weaker social protection, income discontinuity, etc.), while not being subordinate to any employer. On a strictly legal point of view, they are all employees of the BEC and, consequently, may benefit from the social protection that exists in France for employees: pension, invalidity, maternity leave, etc. Indeed, social protection is a major issue as it constitutes the basis of such a model. Within the BEC landscape, the oxymoron “collective entrepreneurship” is a new category used by social actors in opposition to “individual entrepreneurship,” meaning that being a freelance worker does not necessarily require one to work alone.

However, BECs develop different economic and political views. For instance, the first BEC conceives business in a way that does not fit with the “social philosophy that aims to make a ‘self-entrepreneur’ out of everyone;” their goal is to help people to create their own business while protecting them from the difficulties of self-employment (Bureau and Corsani, 2018, p. 285). BECs with the same (or similar) ideological worldviews tend to promote a vision of business based on reflexive actions, aiming to think through social and political issues (internal and collegial democracy, precariousness, work-leisure balance, gender equality, etc.)^15^. The most emblematic examples of such viewpoints are probably Coopaname (France) and Smart (France, but linked to its parent-company in Belgium), which develop intellectual activities and knowledge through action-research, seminars, semi-academic activities (almost “think tanks”), and more^16^. For instance, Coopaname intends to reintroduce the “mutuality” principles^17^ and conceives the cooperative as a “shared company” which is not only composed of aggregated entrepreneurs but that also takes into account the way their activity produces a common value that might then be socialized (Veyer and Sangiorgio, 2018). Coming from a different perspective, some BECs highlight the way in which they boost the achievement of individual entrepreneurial projects or the added economic value they generate for the area. Although solidarity and community are almost always mentioned as important values of such organizations, they do not insist on the need to create either cooperative and self-organized entities or collective rules enacted through democratic decision-making processes led by the salaried entrepreneurs themselves. These BECs are not that different from an umbrella company. The BEC landscape is therefore polarized between, on one side, the idea of an “aggregated entrepreneurs' company” and, on the other side, the quest for “alternative narratives on work and employment” (Veyer and Sangiorgio, 2018, p. 62). This polarization is revealed by the issue of the ownership of wealth^18^, and is, for example, reflected in the way each BEC conceives economic risks, develops the pedagogy of financial management toward its members, and does or does not promote its membership (Veyer and Sangiorgio, 2018, p. 61).

However, in spite of the wide range of political orientations within the BEC landscape, by associating autonomy with social protection (Veyer and Sangiorgio, 2006, p. 92), BECs are considered by some actors and analysts as emancipatory projects, innovative institutions, and “instituting factories” (Bureau and Corsani, 2018) through which workers invent new rights, collective action, and cooperation rules. The project managers as well as the salaried entrepreneurs generally all consider—although in their own way—these organizations to be tools for emancipation, whether they aim to re-create continuity in freelancers' careers, offering new pooled and cooperation spaces, or simply help to achieve entrepreneurial dreams. In short, BECs are thought of as tools to emancipate individuals from a former condition that they do not want anymore (salaried job, autonomous but precarious work, isolated self-employment, bogus self-employment, etc.).

Two major consequences should be highlighted. First, if BECs are “instituting factories,” it seems important to underline the process through which this is done. In their research, French authors Bureau and Corsani study the renewal or invention of new institutions in the gray zones of work and show how initiatives, such as the BEC “borrow from” other traditions (trade unions, cooperatives, etc.) and “recombine them in various ways” (Bureau and Corsani, 2018, p. 293). Indeed, the BEC rests on an old model, the cooperative, that first appeared during the nineteenth century and was legally instituted in France under the status of Scop—*Société coopérative et participative*. This model was reinterpreted in 1995 in France when the first BEC was set up and, later, when the status of salaried entrepreneur was institutionalized through the Hamon law of 2014. So, the BEC uses existing organizational and legal frameworks—the Scop and the standard indefinite employment contract—in order to follow current trends and aspirations: autonomy at work, self-employment, and the ability to combine several jobs during one's life (instead of remaining in a wage-earning job under long-time employment). In other words, BECs “hijack” pre-existing rules^19^, i.e., those of the indefinite long-term contract, which are now interpreted and used in order to give freelance workers salaried working conditions. To follow further on the same lines, we interpret the creation of the BEC through a process of “conversion” of the wage-labor institutions. “Conversion occurs when rules remain formally the same but are interpreted and enacted in new ways. This gap between the rules and their instantiation … is produced by actors who actively exploit the inherent ambiguities of the institutions. Through redeployment, they convert the institution to new goals, functions, or purposes” [(Mahoney and Thelen, 2010), p. 17–18]. This conversion of existing rules—“working with existing materials to craft solutions to new problems” (Mahoney and Thelen, 2010, p. 17–18)—also occurred when BECs had to adapt their rules to the evolution of French labor law, which required, for example, having staff representatives. The BEC tried to do this even though the subordination relationship of salaried entrepreneurs is not the same as the situation of employees in traditional companies^20^. Another example of such adaptations is the use of the employment contract: the working time (full-time, half-time, etc.) is the main adjustment variable that enables both the BEC staff member and the freelance worker to mutually agree to adjust the latter's wage to his/her volume of activity. Since French labor law prevents employers from lowering hourly rates, i.e., wages, the BEC then uses the flexibility of the law to support the uncertainties of self-employment.

**Figure 1:**
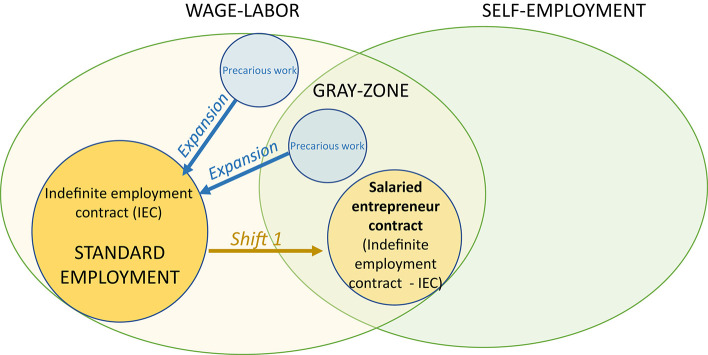
The first type of shift between standard and non-standard work: the indefinite employment contract is introduced into a gray zone in contrast with the classical phenomenon of expansion of precariousness in standard work.

Second, and as a consequence, BECs drag the standard employment relationship of wage labor into a gray zone of employment (see Figure 1). We therefore observe a complete reversal of what usually occurs, that is to say an expanding influence of non-standard employment relationships—often accompanied by precariousness—in standard labor markets^21^. Of course, some criticisms are made of this type of economic model because of its potential ambiguity toward precariousness. Some observers note that Smart enables enterprises, such as Deliveroo to not hire workers, who therefore remain self-employed instead of being reclassified as employees (Drahokoupil and Piasna, 2019, p. 7 and 39). However, BECs generally appear as a kind of inverted-mirror configuration of bogus self-employment and uberization. While the latter implies subordination at the same time as assuming autonomous worker conditions and status, the BEC provides genuine autonomy to freelancers under a salaried status. We may think of salaried entrepreneurs as “false wage-earners” because they consider themselves to be self-employed and yet they receive a pay slip.

This approach “makes gray zones themselves the issue and terrain of a radical, alternative process of institutionalization” (Bureau and Dieuaide, 2018, p. 273), of new rights, political visions, and uses of employment frameworks. The way actors “put the markets to the test” (Bureau and Dieuaide, 2018, p. 268) also advocates for a concrete and micro-scale analysis of political changes in the workplace. Such diversity and openness within the gray zones suggest that working and employment conditions also result from the creativity and interplay of objective frameworks and their subjective appropriations by social actors. However, this is not sufficient to understand the meaning of gray zones, and one may wonder what the effective practices are behind this framework. In fact, neither organizations or statuses, nor standards of employment are stable, and determined entities and their functions, effects, and meanings are also produced by the people who invest in them. On an individual level, it seems important to bear in mind the degree of freedom that actors always practice within the framework of institutions, and the heterogeneity of subjectivities and experiences in the workplace and within the course of one's working life. How is the “hijacked” indefinite employment contract used and experienced by entrepreneurs? What are their dreams, ambitions, and representations of “emancipated” working conditions?

### When the Hijacked Contract Is Hijacked Again: the Diversity of Career Pathways, Aspirations, and Political Values of Salaried Entrepreneurs

Some indigenous systematic associations are widespread in society by a number of actors including political leaders, the media and, of course, our interviewees. For example, they tend to lie together entrepreneurship and individual emancipation or freedom. On the opposing side, wage labor is commonly represented as a protective but also alienating status as it counters the individuals' autonomy at work and is based on economic exploitation. Instead, in order to analyze the complexity and transformation of work, we assert that we should now pay attention to the disconnection between legal statuses, social identity, and actors' practices. This phenomenon is widespread: for instance, one may be an autonomous employee (Grégoire and De Heusch, 2016), which means that in spite of being a wage earner, one may consider oneself to be a “freelance” worker from a subjective point of view and, de facto, manage one's activity (work content, skills and knowledge, schedules, and professional/private life balance). This is the case with “project-based” work. A further example is an employee who may also have a part-time job that he/she carries out as a self-employed person; even if this job does not provide significant income, it represents a major identity anchor because it is through the job that the worker defines him/herself. This is the case for many creative and artistic workers, for example, who consider themselves to be a musician or a painter even though they are a teacher for the majority of their working hours (Perrenoud, 2007). On the opposing side, one may be a self-employed worker from the legal point of view but economically dependent and deprived of any autonomy in the workplace, such as bogus self-employed people. If we apply this to the case of BECs, one wonders what is meant exactly by working as a salaried entrepreneur? What is the aim of social actors who endorse such a status? In what range of diverse conditions do they work and what would represent ideal working conditions to them? What are their worldviews, dreams, and ambitions?^22^

The way the interviewees appropriate the same situation in different ways—the CAPE or CESA contract, or former professional status, such as that which they experienced as temporary agency workers, for instance—are major incentives to further explore work experiences. A single term (“wage earner,” “entrepreneur,” “temporary agency worker,” etc.) may have a wide range of meanings. In this article, we show how categories are therefore not sufficient to analyze social phenomena *per se* based on the diversity of conceptions we observed during our fieldwork. In other words, it is necessary to consider the “double reality of work,” as described by Bourdieu (1997), that is to say both the objective dimension of work and the subjective perceptions of it. The notion of “praxis” seems relevant to understand the mechanisms of such appropriations in a qualitative approach. In order to do this, one has to consider people's social pathways and situations in depth which requires a qualitative approach. This is why we have been elaborating an analysis framework which enables us to focus on the longitudinal aspects of people's careers, as well as the social diversity of their lives. Through these tools, we were able to analyze our interviews in a systematic way through the two dimensions of the praxis that finally covered the following aspects: work, living conditions and conflictuality, i.e., the consciousness of divisions that social actors possess and where they position themselves in social stratification, as well as the generalization they are able to make about social issues.

Through these criteria, we were able to systematize the rich and abundant data produced from each interview. Several tendencies emerge and what follows are some examples of the diversity of views and practices developed by an individual. These appropriations on an individual level therefore involve a second type of “shift” in the original rules of the standard employment contract.

**Jean-Philippe**^23^ is a plumber in his late thirties who comes from a deprived background. Following his mother's death when he was just 2 years old, he was raised by his father, a boilermaker in a poor suburb of the city. After his father died, Jean-Philippe, then 17, spent the next 3 years in a children's/young person's institution. As he was not allowed to stay there once he turned 21, he had to find a way to earn a living very quickly, and he passed a 2-years course to become a plumber. He then experienced the wage-earning status between the ages of 20 to 37, mainly through temporary agency work. As he was an excellent plumber, he was very highly rated and could earn around 1,900€ per month. In 2015, he therefore decided to further develop his career (in terms of salary and skills) but, unlike most workers, he rejected the idea of obtaining a long-term contract in a company: first, because he considers bosses to be “crooks” making profit by underpaying their employees, and second, because he thinks that the competition between employees would have made it difficult for him as he considers himself to be a “big mouth.” He also justifies this atypical position toward the standard employment contract by a fear of “getting bored and rusty.” However, he did not want to become genuinely self-employed either because he does not feel comfortable with business relations and communication skills. He eventually discovered the BEC model and entered the cooperative in 2017 under a CAPE contract. Jean-Philippe is not interested in the cooperative for the ethical values it promotes nor for its internal democracy; he does not even differentiate between a BEC and an umbrella company. However, he is very happy with the autonomy he obtains through it, as well as the security and advantages it offers: a professional network, paperwork being done by the cooperative, and unemployment benefits as well as the ability to make unemployment benefit contributions for future eventualities. As he is a lone parent and is aware of life's difficulties, he cares very much about being “responsible.” This means not only doing good work but also thinking about his entrepreneurial strategy in both the medium and long term, for example by avoiding undeclared work as much as possible. In fact, Jean-Philippe relies upon his own efforts to succeed. He despises politics even though he has clear political views, for example denouncing the stigmatizing and racist views toward inhabitants of the suburbs that are presented by what he calls the “television democracy.” For the past 18 months, under the CAPE contract, he has therefore earned a living by combining unemployment benefit with the reimbursement of his expenses by the cooperative (as wage earners are allowed to do within any company in France). In parallel, the income he obtains from his work is deposited in his personal account in the BEC; he thus hopes to become a salaried entrepreneur and sign a CESA contract within the next 6 months. Jean-Philippe's clients are of two types: the first are individuals who ask him to carry out plumbing work in their homes; the second is Jeremy, another member of the BEC, who works as a mason and sometimes asks Jean-Philippe to do the plumbing aspects of his work. He is thus Jeremy's sub-contractor, while maintaining strong autonomy in how he does his job as well as in deciding the amount that he invoices Jeremy for. As his business now works well, Jean-Philippe is also seriously considering the possibility of using sub-contractors himself, for he explains that some members of the cooperative work as laborers for other members. He uses the term “solitary worker” to define himself: he says that “who leads in the cooperative [the CEO] does not need to be discussed” but does not consider himself to be a subordinate worker, or a “boss,” or an “entrepreneur.” The case of Jean-Philippe thus illustrates the different enshrined subordination relationships in which a freelancer may work and the unstable subjective and objective positions associated with this position.

**Maïwenn** is in her mid-fifties and works as a therapist in “non-violent communication.” Maïwenn divides her job into three aspects: non-violent communication instructor, therapist, and consultant for health institutions. She settled in the city 5 years ago after a long career as a salaried worker, during which time she was a project manager in the Paris fashion industry for almost 20 years. After she realized that she did not wish to continue with that way of life—a well-paid job but one that did not fit with her ethical values—she decided to quit and retrain. She became a real estate agent in the south of France countryside while simultaneously learning the principles of “non-violent communication.” She then started a 5-years training program to become a specialist and eventually became a “non-violent communication” therapist as a member of a BEC. During this time, she also continued working as an employee of a real estate agency in Paris. After a couple of years, she could not manage both jobs and therefore quit her freelance activity in the BEC. When she settled in the city, a year and a half ago, she ended her real estate agent job and employee status and became a freelancer in a second BEC, which then collapsed. Consequently, as a salaried entrepreneur, she was dismissed from this first BEC for economic reasons (2 months before the interview) and could then obtain unemployment benefits. At the time of the interview, the benefits were contributing to her income while she was working under a CAPE contract in a third cooperative. She needed to accumulate income (i.e., money coming in) in her personal BEC account in order to become a salaried entrepreneur again in the new BEC. She says that her income nowadays is “probably divided by three times” in comparison with her former income in the luxury fashion industry. Now, she considers herself an “entrepreneur,” in the sense that the term refers to the ability to comply with a wealth of tasks that freelance work requires—finding costumers, advertising, social media, management of relationships with clients, etc.—although she feels she lacks computing and social media skills in particular. However, unlike Jean-Philippe, who feels relieved that the cooperative is in charge of such tasks, Maïwenn's principal motivation to become a member of a cooperative is the possibility of doing training services as an instructor through the BEC's training agreement. Of course, she is very eager to participate in the BEC's activity as she is highly interested in the social and solidarity economy sector and democracy in cooperatives. She conceives the BEC as an entity that each member should be responsible for and she places a high value on individual wisdom to create an optimal organization (she describes herself as a “utopist”). She rejects the idea of hierarchy, competition, and control inside organizations (referring to *Manufacturing Consent* by Chomsky). Fundamentally, Maïwenn considers herself to be an autonomous worker, whatever her legal status. Throughout her entire career, her position in work power-relationships have always given her the opportunity to control her schedules and activity. Influenced by the “anarchist ideas” of her family background, she says: “I had been an employee for years, de facto, but in my mind, I never ever positioned myself as such.” For example, she refuses to call the CEO of the cooperative a “boss” and rejects the idea to “put [herself] in any asymmetrical relationship.” She does not make a point of being self-employed or an employee: she endorses her position as a member of a cooperative because she could not hope to carry out the three types of activity that she does if she worked as an employee in a single organization. Maïwenn is a perfect example of a pluriactive freelancer who, above all, cares about his/her personal autonomy regardless of his/her status. As she says, “The framework is just a tool; what matters most is what one does inside of it.”

**Pierre**, who is in his late fifties, settled in the area two and a half years ago, and works as a consultant in enterprise business strategy. At the time of the interview, he had been self-employed in this work area for 18 years and had joined the cooperative 6 months earlier, directly under a CESA contract, that is as a salaried entrepreneur. After Pierre obtained his political science degree from a selective university in 1984, he started working in public institutions and insurance companies. Apart from a single attempt to become a self-employed consultant during the 1990s—a brief experience which failed—he was an employee for 16 years, working as an expert in entrepreneurship and small businesses. In 1998, he started work in an insurance company in which he discovered a new organizational model based on horizontal governance and employees' individual skills and responsibility; he still considers it to be an “extraordinary experience” that profoundly changed his philosophical views on organizational rules in companies. At the beginning of the 2000s, he quit his salaried position and became a self-employed consultant, a job that he has been doing in the center of France for almost 18 years. During that time, he has never accepted the auto-entrepreneur status, as he would earn too much money (between around 120,000€ and 150,000€ per year, he says, which represents a minimum of 10,000€ of monthly gross incomes, and therefore, an average of 4,000€ of monthly net revenue). Pierre does not fear major life changes and fits the image of the flexible and adaptable entrepreneur. He describes his life as “non-linear,” including two marriages, some variations in his income, a move to the south of France, and several working experiences. For instance, 3 or 4 years ago, he decided to implement a new idea which involves software that enables small companies to better define their marketing strategy in relation to their networks. In order to implement this idea, Pierre devoted less time to his traditional activity of consultant, and began dedicating time and money to his new invention (for example, he sub-contracted the technical development of the software). Like Maïwenn, making his dream come true resulted in a substantial loss of income of around 50% to 60%. He represents the typical figure of the entrepreneur who climbed the social ladder due to his taste for risk and innovation and he even considers himself to be an employer at times. He is very critical of employers' unions (“that kind of stuff pisses me off”) because he believes that they are not really able to take effective action. However, he appreciates French president Macron's position, which he finds “innovative,” and he frequents pro-Macron circles in the city. At the same time, Pierre does not feel at ease socializing with “high-ranking” people, and finds this “stilted.” He also asserts that the first aim of any business should be human well-being, not profit. Pierre comes from a working-class family—his mother is a housewife and his father is a subordinate worker in a big French company—and this might explain why he places so much importance on social values. For instance he makes it a point of honor to decline business partners who neglect their employees' working conditions and security. The human dimension and innovative models of enterprise—based on horizontality and strategic networking—are two of his main concerns. He strongly dislikes a “slapdash” organization: this is one of the criticisms he makes of the cooperative to which he belongs and is why he wishes to commit himself further to building it up. “Network” is Pierre's leitmotiv as not only is it the core concept of his invention but it was also his first motivation to integrate a cooperative: when he arrived in the city in 2014, he suffered a lot from loneliness, especially at work. In a nutshell, Pierre is not interested in the cooperative model from a strictly legal point of view—the share of the labor value—but he thinks that “collective entrepreneurship” (as opposed to the traditional model of individual entrepreneurship) is the future of small businesses in France.

Jean-Philippe, Maïwenn and Pierre are representative figures of solo self-employed people in France at the current time: a plumber who illustrates the traditional highly skilled manual worker, a category that has declined in France over the last two decades [from 46.9% of the self-employed in 1994 to 34.3% in 2014 (Jansen, 2016, p. 6, based on Eurostat data and the EU Labour Force Survey (1994, 2004, 2014)]; and two professionals who embody the business and other service sectors that have been expanding very significantly [from 18.7% in 1994 to 34.2% in 2014 (Jansen, 2016, p. 6–9)]. In classical analyses, the self-employed are assumed to be a relatively homogenous group with shared interests (autonomy at work, costs and rewards of working on one's own account, possible position of employer, entrepreneurial risks)^24^. For some analysts, worker-owners, in general, occupy a new and ambiguous emergent class position, different from that of conventional workers or of the petty bourgeoisie (Allen-Whitt and Rotschild-Whitt, 1986). Here also, our sample of interviewees is, by definition, a very particular sub-type of the self-employed, as they all enjoy great autonomy at work. To that extent, they cannot offer a viable generalization of other sub-types of the self-employed, especially those who experience dependent relationships with a single contractor, for instance.

About the similarities shared by our interviewees, and beyond these three cases, the widespread conception that administrative tasks are “dirty work” (Hughes, 1971) is shared by all our interviewees (because they feel they lack competency or that admin wastes time at the expense of their core activity). The recourse (or absence of recourse) to social rights and protection is relatively similar: the overwhelming majority of interviewees think about their unemployment benefits but often overlook other rights (maternity leave, health and safety, paid holidays, etc.) or do not mention them spontaneously. Alongside these common trends, it appears that interviewees do not distance themselves from the wage-labor contract *per se*, just as they are not attracted by self-employment in itself: people are actually able to identify the advantages of one or other of these situations (e.g., unemployment benefits and the social protection of an indefinite employment contract) and devise strategies to combine them. This finding thus contradicts political discourses asserting that people nowadays tend to reject wage-labor work and aim to enter entrepreneurship. Considering these elements, most salaried entrepreneurs seem close to the “ascending figure” of the gray zones, “associated with a quasi-militant approach to the recognition of new work relationships that are not dependent on subordination (e.g., the figure of the hacker, or that of the entrepreneur-employee)” (Bureau and Dieuaide, 2018, p. 267, quoting Azaïs 2016). However, some fragmentations appear: in more recent studies, the heterogeneity of the self-employed has been investigated by researchers. Here as well, through these three case studies it is interesting to observe the variations within that single sub-type of self-employment. They remind us of re-fragmentations, for example between “genuine self-entrepreneurs and subordinates” and “formal and informal sectors”) observed in other gray zones, such as auto-entrepreneurship in Brazil (Rosenfield, 2018). They also advocate for alternatives to analytical oppositions between “entrepreneurship” and “precariousness” in the gray zones of work (Murgia and Azaïs, 2019). To that extent, our study converges with other findings which relativize the image of the self-employed as a homogeneous social class.

From this perspective, introducing the political dimension of work is useful. From a qualitative approach, the notion of “figures” proposed by Azaïs is interesting as it “makes it possible to explain the heterogeneity of the positions of actors in gray zones. In a manner of speaking, these figures represent a spectrum of ambiguous positions with regard to labor and employment institutions. They are positions that can be understood as being situated along a ‘constraint vs. freedom’ axis, depending on whether or not workers are forced to take such work, whether they accept it, defend it or even outwardly advocate it and act to bring about the new situation” (Bureau and Dieuaide, 2018, p. quoting Azaïs 2016). With different methodological views, Jansen's statistical analysis of the recent changes in the occupational and sectoral structure of self-employment in Western Europe show that “politically the self-employed are more heterogeneous than traditional class-based theories assume” (Jansen, 2016, p. 23). In his work, the “constraint vs. freedom” axis is also present through the notion of autonomy at work^25^ and job insecurity, both used to explain the political orientation of the self-employed in Europe. “People in solo self-employment are generally more likely to support welfare policies and (new) left parties—and oppose right-wing parties—as they are more insecure with respect to their income and/or job. … Economic vulnerabilities might challenge the archetypical image of people in self-employment as an economic conservative, political right-wing class. This observation suggests that particular segments of self-employment may share the characteristics of other forms of ‘atypical’ work, not only with respect to labor market insecurities, but also regarding the political orientations associated with such insecurities” (Jansen, 2016, p. 22). To that extent, it is clear that our findings corroborate these sociological studies. Here, the “freedom vs. constraint” axis is also present at different levels, especially through two criteria:

- “working conditions”: for instance, some earn a living by combining unemployment benefits and working under an indefinite employment contract—CESA—, while others are already financially stable and receive a comfortable wage.
- “position within power relations in the workplace”: some are contractors, others sub-contractors; some have experienced “autonomous” work even though it was under an employee contract, while others know well what “having a boss” means.

First, an important finding is that in addition to objective conditions, the meaning that interviewees give to terms, such as “boss,” “autonomy,” “entrepreneur,” “wage labor,” “self-employment,” “cooperation,” etc., is varied: in fact, these categories do not have a meaning *per se*. The in-depth interviews show that the interviewees give a different meaning to a single term. In particular, this depends on their past and social pathway, and the “freedom vs. constraints” axis needs to be considered as a longitudinal approach as this analytical framework fully explains most of freelancers' current representations. As we observed through the case studies, past experiences are decisive in understanding current conceptions that interviewees have of themselves: considering oneself as an “employer” or a “subaltern worker” sometimes means experiencing such positions, although not necessarily currently. This point therefore challenges the issue of identities at work and requires further investigation.

Second, an objective constraint may be viewed negatively or positively depending on the individual, for instance Jean-Philippe, who very much appreciates agency work for the freedom it provides, while avoiding the “dirty job” of administrative tasks. However, this form of work is generally considered precarious. Another example is the cooperative' rules. They may be perceived as constraints imposed from the top down that a member may respect (and at the same time avoid or circumvent): for instance, doing some informal work but not too much. Or they may be thought of as necessary and even desirable elements that members will go along with because they make the collective entity viable. This perception of rules and objective constraints also depends on people's relationship to *cooperative ethical values*, i.e., caring and knowing (or not) about them.

**Figure 2:**
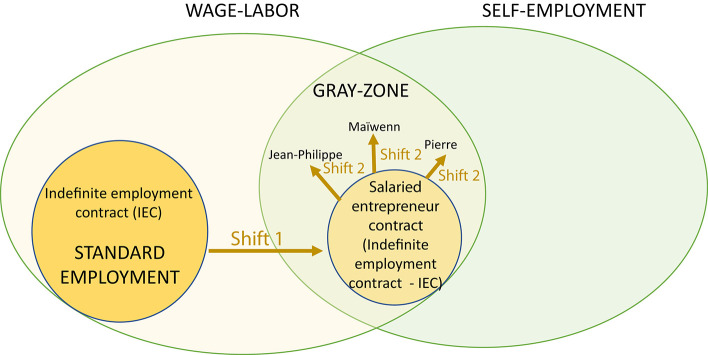
The second type of shift occurs when individuals adapt the indefinite employment contract to their own entrepreneurial strategy.

More generally, the difference between individual positions toward categories and objective constraints is caused by the multiplicity of political aspects which can be subsumed into two variables. The first is *political competency* through political ideas (some embrace anarchist philosophy while others promote economic liberal trends) and the “statutory dimension” of political competency [(Bourdieu, 1979, p. 466–479), the feeling one has to be able to position himself/herself on social and collective issues]. The second variable is *conflictuality*^26^: perceiving and situating oneself in social conflictuality appears not only when the interviewees talk about what they experience in the workplace but also through general considerations. This complements Jansen's analysis, showing that the self-employed develop different political viewpoints depending on their position inside the labor market and the risks associated with it (Jansen, 2016). In the interviews, we can see the clear awareness that entrepreneurs have of their position in the social strata and how they adapt their entrepreneurial strategy to combine the rules of the BEC with their own interests (see Figure 2). This also provides complementary explanations to analyses of institutional changes and of the different ways in which social actors fulfill projects and career pathways within organizations. Compromise and compliance between the rules of a BEC and practices of individuals (Mahoney and Thelen, 2010), and margins of action and innovation (Bureau and Dieuaide, 2018, p. 273), are different paths to give substance to these possible ways of fulfilling freelance workers' projects. On a micro-scale analysis, considering different expressions of the political dimension may help us comprehend under which conditions people take these routes of change within institutions.

## Conclusion

Some social scientists tend to automatically conclude that the social and solidarity economy sector is virtuous as they base their analysis on organizational and theoretical rules about the way it is *supposed* to work. This position is the mirror-image of normative approaches, making the gray zones of employment a space of “failures” of labor markets. Both approaches tend to analyze the reality of work as more or less “lacking,” depending on how much it corresponds with the theoretical model. This article shows that analyzing the *practices* of actors is a fertile way to avoid these problems. Of course, it remains important to temper the relativism toward objective frameworks and underline the crucial nature of legal constraints in the workplace. The recent multiple attempts to reclassify the self-employed status of Uber drivers into employee contracts is evidence of the considerable importance of these objective aspects'. Nevertheless, wage labor, especially through the long-term indefinite employment contract, which represents one of the “purest” forms of standard employment relationships, may be interpreted in different ways'. The case of BECs confirms that gray zones are not necessarily chaotic and “although it deviates from what is empirically and normatively considered to be ‘normal,’ nonstandard employment is not necessarily ‘bad’ or ‘precarious”’ (Hipp et al., 2015, p. 367). Rather, this phenomenon appears to be a testimony to the ability of social actors to resist and challenge existing institutions and to invent new ones (Bureau and Dieuaide, 2018, p. 265).

BECs are anchored in a wider context of the renewal of experiences in trying to cope with the transformation of the labor market. Through worker-owned cooperatives, one can better understand the continuity between the renewal of industrial and entrepreneurial cooperativism as both are a common attempt to reposition workers in power relations within a context of increased flexibilization of labor markets. However, the case of BEC ignores the issue of the stability—or the vulnerability—of the position of “salaried entrepreneurs” in the labor market.

With respect to this issue, this article shows how the implementation of such contracts on an individual level creates several ways of experiencing work. So, rather than a “pure” form, we assert that the standard employment contract is nothing but a wide range of concrete—organizational and individual—interpretations. BECs veer between standard and non-standard employment relationships as, at a meso and organizational level, the standard norm is dragged into a gray zone (part 3.1). Then, through multiple negotiations and adjustments that freelance workers make around their employment contract, the meanings of “being member of a BEC” differ (part 3.2). Through the notion of praxis, which provides a systematized analytical framework for the ethnographic data, the political dimension appears as a crucial variable to explain variations between interviewees. We eventually subsume its variant forms through the notions of conflictuality and political competency. Besides, the concept of “social class”^27^ is not outdated and in the gray zones of work, where legal statuses may be separated from identities and practices, it provides a pertinent factor of analysis, enabling actors to situate their own position and define their worldviews.

Regarding these methodological aspects, it is important to point out that the BEC case study introduces complementary qualitative guidelines for other inquiries. However, it is important to remember that this case study does not prevent us from considering a macro-scale analysis. First, this is because BECs remain experiments at a local level, even though they can then be articulated and even intertwined with legal evolutions of State and regional policies. Second, it is necessary to locate actors' practices in deeper trends, reached through statistical analysis.

So, on the one hand, the entrepreneurs enjoy less stability than the traditional figure of the self-employed (agricultural and craft workers, for instance) because, unlike the latter, the “new” self-employed are less likely to remain in this position and will return to an employee status or become unemployed (Jansen, 2016, p. 12, quoting Arum and Müller; Schulze Buschoff and Protsch). This means that being an “insider” of the labor market, due to an indefinite employment contract, does not provide *per se* sufficient stability if other working conditions are not assured. On the other hand, regarding the idea of the “wage society” analyzed by Castel (2002b), a large majority of entrepreneurs have a strong consciousness of the “social property” to which they are entitled due to salaried employment contracts^28^; to that extent, they therefore enjoy a moral and material security that the traditional self-employed lack. In particular, wage labor is considered by interviewees, whatever their political worldview, to be a serious and efficient alternative to the French “auto-entrepreneur” status and, in particular, the salaried entrepreneurs demonstrate a sharp awareness of their rights to unemployment benefits. These elements therefore contradict the idea promoted by public policies that, in the twenty-first century, workers massively seek to become independent and emancipate themselves from the “yoke” of wage-labor.

However, our inquiry also shows that, beyond this common attachment to the wage-labor contract, social actors implement individual and autonomous tactics to use it: entrepreneurs demonstrate precise accumulation strategies that they may implement due to the CAPE and CESA contracts, which enable them to use these benefits in the future if required. Although we may not develop these aspects in the present article, it is important to underline that, depending on organizational strategies and internal governance of each BEC, the training of entrepreneurs in the categories and rights attached to the wage-labor contract strongly differ between one BEC and another^29^. Whereas, in the “wage society,” “the individual in society acquires social citizenship by taking part in the collective benefits and services guaranteed by the state” (Castel, 2002a, p. 328), our interviewees develop an ambiguous position toward it. The choice to work in a BEC does not result from philosophical and political expectations originally linked to the principles of the wage society, such as the reinforcement of interdependence between the members of society, the promotion of jointly held goods, etc. Rather, the promotion of the wage-labor contract and belonging to a BEC results, for many interviewees, from the individual consciousness of its benefits in terms of rights and personal protections. This articulation between these subjective worldviews and attachment to this objective framework is another area for further investigation.

Finally, does this “conversed” classical wage-labor contract remain a “standard” form of employment? Answering such a question requires to point out the impossibility of classifications. Furthermore, we make the assumption that there might be nothing but “hijacked,” particular, altered, and creative practices, coexisting alongside other “hijacked,” particular, altered, and creative practices. Using and re-interpreting legal frameworks is not a specificity of BEC, and other types of contracts are used flexibly, with a margin of maneuver, or are subverted by social actors until the law endorses adjustments, these two phases being a constant and circular process, such as with flexible work in France (Kornig, 2003, p. 111–112). The issue of “purity” of labor and employment contracts, and the way it gives way to successive conversions, refers to the legal distinction between the law and the customs of law. From a philosophical and anthropological perspective, and to return to where we began this article, we quote the philosopher Bachir Diagne: “There is not an already constituted universality, with the stability of a *telos* overlooking, from its own self-assured exemplarity, anthropological proliferation and fluctuation” (Bachir Diagne, 2013, p. 16). Like this supposed pure universality opposed to its cultural variations, we argue that there is no “properly used” wage-labor employment relationship of which the opposite would be a “degraded form.” From this conception, the salaried-entrepreneur contract is just one manifestation of the wage-labor contract, which is a precise and constraining, but nevertheless flexible, “signifier” whose meaning and content would depend on actors' actions.

## Data Availability Statement

The datasets generated for this study will not be made publicly available. All qualitative data were collected on condition of anonymity. Requests to access these datasets should be directed to Flora Bajard, flora.bajard@gmail.com.

## Ethics Statement

Ethical review and approval was not required for the study on human participants in accordance with the local legislation and institutional requirements. Written informed consent for participation was not required for this study in accordance with the national legislation and the institutional requirements. Written informed consent was not obtained from the individual(s) for the publication of any potentially identifiable images or data included in this article.

## Author Contributions

The author confirms being the sole contributor of this work and has approved it for publication.

## Conflict of Interest

The author declares that the research was conducted in the absence of any commercial or financial relationships that could be construed as a potential conflict of interest.

## Footnotes

^1^Defined as “full-time, indefinite, as well as part of a subordinate and bilateral employment relationship” (International Labor Organization, 2016, p. 7).

^2^The ILO defines four types of non-standard work: temporary, part-time, agency, and economically dependent work.

^3^For example, in Western Europe, where social protection is intertwined with employment status—and the salaried status in particular—the separation between the two categories has generated a two-sided social protection system (which is based on different insurance funds).

^4^In terms of employment in the second labor market and living conditions. Regarding the diversity of empirical situations and narratives about non-standard work, see for example: Hipp et al. (2015).

^5^Our translation.

^6^“Precariousness” is a sociological category that deserves discussion but, in line with Cingolani, in this article it refers to the discontinuity of times and activities within socialization processes imposed unilaterally by managerial and productivity discourses and actors, i.e., a loss of autonomy and a new form of subjection (Cingolani, 2008, p. 109–111).

^7^Gray zones are often considered a regression in comparison with the standard employment relationship (see for example Bisom-Rapp and Coiquaud, 2017, p. 2).

^8^See the work of Bureau and Dieuaide (2018).

^9^We use the term “freelance worker” in this text because the workers we are talking about are not real “self-employed” but belong to a cooperative as salaried workers (part 1). We also avoid the term “entrepreneur” as it conveys particular indigenous—political and economic—values. However, “entrepreneur,” “autonomous worker,” “freelance,” and “solo self-employed” used in different contexts may describe very close—or even similar—situations; a single term may also mean different things depending on the speaker (part 2).

^10^According to the data provided by the two BEC Federations, Copéa and Coopérer pour entreprendre: https://www.copea.fr/index.php/qui-sommes-nous/notre-histoire; https://cooperer.coop/.

^11^Oral informed consent was obtained from the participants of this study.

^12^Decree n°2015-1363 of October 27, 2015 on Business and Employment Cooperatives and salaried entrepreneurs.

^13^This is in addition to a percentage—generally between 10 and 15%—of their sales that each entrepreneur gives every month as a financial contribution toward the operation of the shared organization.

^14^For more details about these three steps, see Bureau and Corsani (2018, p. 285–286).

^15^For an historical and precise analysis of this stream within the BEC movement, see Bureau and Corsani (2018).

^16^The BEC also represents the renewal of intermediary bodies, as some of them are legal advisers, employment intermediaries (e.g., the Belgian cooperative Smart through its agreement to hire Deliveroo riders between 2016 and 2018), and also represent workers on a political level (e.g., Smart takes part in collective bargaining on self-employed workers conditions on a European level, along with the European Confederation of Trade Unions).

^17^For details about emancipatory projects based on the Coopaname case, see Bureau and Corsani (2018, p. 286).

^18^Sangiorgio and Veyer propose an in-depth analysis of this issue within BECs and suggest an essential and enlightening difference between employment (the status that each freelance worker has, associated with a physical individual) and activity (autonomous units of production). Some BECs confuse these categories, which leads to the BEC acting as a support for individual projects and an aggregation of entrepreneurial businesses, whereas others, by distinguishing both notions, make it possible to think through the added-value of each activity that will or will not be socialized.

^19^Bureau and Corsani also interpret this phenomenon as a form of “hacking” of the institutions of wage labor (Bureau and Corsani, 2018, p. 289).

^20^See Bureau and Corsani (2018, p. 292) and Devolvé and Veyer (2011).

^21^For example, the situation—and mobilization—of Deliveroo bikers or Uber drivers.

^22^Emancipation, as an experience or an aspiration, and as *freeing from* a former condition, but also as exercising a *genuine self-reliance* (Berlan, 2016; Jacquot et al., 2019), actually refers to a wide range of practices and discourses among the people we interviewed (see below).

^23^The names of the interviewees have been changed to protect their anonymity.

^24^See a state of the art in Jansen (2016, p. 2).

^25^Autonomy is a criteria widely used by analysts to observe self-employed political behavior: see for example see (Jansen, 2016, p. 11, quoting Kitschelt and Rehm 2014; Kohn 2015). However, Jansen shows that the degree of autonomy does not determine significantly political orientations of the self-employed (Jansen, 2016, p. 22), unlike economic vulnerabilities: “insecure self-employed workers tend to support new-left parties. … [but] the right-wing political orientation of self-employed workers is not stronger as they have more autonomy over their job” (Jansen, 2016, p. 19).

^26^More than to critical discourses *per se*, the expression of divisions related to social issues, as well as the generalization about social topics, constitute the basis of ordinary politicization, according to French politist (Leca, 1973), reinterpreted later through interactionist framework (Hamidi, 2006). This analytical framework is also ours, for it enables us to comprehend ordinary political phenomena far beyond the strict idea of political competency that relates to particular and restrictive skills and knowledge (Blondiaux, 2007).

^27^At the first stage of our fieldwork, aiming to comprehend the relationships to wage labor and self-employment, we were not able to identify a need for racial or gender variables in the analysis. The people we met did not show signs of racialization; gender is probably more likely to be considered at a deeper analysis level to better understand issues such as working conditions, income, and the balance between working and private life.

^28^Social property is, “on the one hand, generalization of the use of jointly held goods, which can neither be appropriated individually nor marketed, and which are in the service of all; on the other hand, generalization of personal protections constituted on the basis of work and no longer on the basis of a private patrimony” (Castel, 2002a, p. 328).

^29^Some BECs resemble “umbrella companies” and provide almost no training regarding “accidents at work,” social insurance and sharing of the risks, history of cooperatives and societies of mutual solidarity, etc.; other BECs are considered as political alternatives to the neo-liberal philosophy and also aim to make their members more conscious and committed to the construction of a commonly shared worldview regarding these aspects.
